# Therapeutic targeting using tumor specific peptides inhibits long non-coding RNA HOTAIR activity in ovarian and breast cancer

**DOI:** 10.1038/s41598-017-00966-3

**Published:** 2017-04-18

**Authors:** Ali R. Özeş, Yinu Wang, Xingyue Zong, Fang Fang, Jay Pilrose, Kenneth P. Nephew

**Affiliations:** 1 Molecular and Cellular Biochemistry Department, Indiana University, Bloomington, IN 47405 USA; 2 Medical Sciences Program, Indiana University School of Medicine, Bloomington, IN 47405 USA; 3 Indiana University Melvin and Bren Simon Cancer Center, Indianapolis, Indiana 46202 USA; 4 Department of Obstetrics and Gynecology, Indiana University School of Medicine, Indianapolis, IN 46202 USA; 5 Department of Cellular and Integrative Physiology, Indiana University School of Medicine, Indianapolis, IN 46202 USA

## Abstract

Long non-coding RNAs (lncRNAs) play key roles in human diseases, including cancer. Functional studies of the lncRNA HOTAIR (HOX transcript antisense RNA) provide compelling evidence for therapeutic targeting of HOTAIR in cancer, but targeting lncRNAs *in vivo* has proven to be difficult. In the current study, we describe a peptide nucleic acids (PNA)-based approach to block the ability of HOTAIR to interact with EZH2 and subsequently inhibit HOTAIR-EZH2 activity and resensitize resistant ovarian tumors to platinum. Treatment of HOTAIR-overexpressing ovarian and breast cancer cell lines with PNAs decreased invasion and increased chemotherapy sensitivity. Furthermore, the mechanism of action correlated with reduced nuclear factor-kappaB (NF-κB) activation and decreased expression of NF-κB target genes matrix metalloprotease 9 and interleukin 6. To deliver the anti-lncRNA to the acidic (pH approximately 6) tumor microenvironment, PNAs were conjugated to pH-low insertion peptide (pHLIP). Treatment of mice harboring platinum-resistant ovarian tumor xenografts with pHLIP-PNA constructs suppressed HOTAIR activity, reduced tumor formation and improved survival. This first report on pHLIP-PNA lncRNA targeting solid tumors *in vivo* suggests a novel cancer therapeutic approach.

## Introduction

Non-protein coding RNAs make up the majority of the transcripts in the genome and both small ncRNAs (<200 nucleotides) and long non-coding (lnc) RNAs (>200 nucleotides) are considered to be functionally important. LncRNAs form distinct secondary structures enabling key interactions with DNA, RNA, or multi-protein complexes^1^ to regulate mRNA transcription and translation, bridge proteins to chromatin, serve as molecular decoys, and guide chromatin modifying enzymes to specific genomic loci to coordinate multiple biological pathways^2^. Furthermore, aberrant lncRNA expression correlates with multiple diseases^3^, and evidence is evolving that lncRNAs play critical roles in tumor progression and metastasis^4^. In this regard, HOX antisense intergenic RNA “HOTAIR” is frequently overexpressed, promotes metastasis and is predictive of decreased survival in a range of cancers and leads to homeotic transformation^4–8^. HOTAIR contributed to cellular senescence by transcriptionally activating NF-kB during DNA damage and increasing Interleukin-6 (IL-6) and matrix metalloproteinase 9 (MMP-9) expression in platinum-resistant ovarian cancer^9^, making it a potential therapeutic target in ovarian and other cancers.

There is increasing focus on the clinical application of lncRNAs as potential biomarkers and therapeutic targets in diverse cancers^2, 4, 10^. However, due to thermal instability, sensitivity to ribonucleases, and inefficient target tissue delivery, recent approaches for tumor targeting of lncRNAs using siRNAs and locked nucleic acids (LNAs) have been met with limited success^11, 12^. Peptide nucleic acids (PNAs) are resistant to nucleases thermally stable, contain a neutral charged peptide backbone that can be conjugated to nucleic acids, and can be modified for *in vivo* applications^13^. Cheng *et al*., used sequence-specific PNAs that bind to target microRNAs to demonstrate tumor-specific targeting of non-coding RNA has-miRNA-155 in lymphoma^14^, providing proof-of-concept for PNA-mediated delivery of noncoding RNAs in human disease.

In this report, we developed a PNA-targeting strategy for HOTAIR serving as a scaffold for polycomb repressive protein complex 2 (PRC2), PRC2 enrichment at specific loci, trimethylation of histone H3 lysine K27 (H3K27me3) by enhancer of zeste 2 (EZH2) and subsequent gene repression^5, 15^. We demonstrate that PNA targeting of HOTAIR RNA single stranded regions^16, 17^ effectively blocks the HOTAIR-PRC2 interaction, inhibits ovarian and breast cancer cell invasion and re-sensitizes to chemotherapy via NF-κB activation and secretion of IL-6 *in vitro*. The “anti-lncRNA” agent decreased ALDH1A1 activity in ALDH(+) ovarian cancer cells, suggesting HOTAIR inhibition with PNA could reduce the ovarian cancer stem cell population. Conjugating PNAs to pH-low insertion peptide (pHLIP) allowed for PNA-targeting to the *in vivo* tumor microenvironment, circumventing lower tumor pH levels due to oxidative phosphorylation^18^. In mice harboring platinum-resistant ovarian tumor xenografts, pHLIP-PNA treatment resensitized and reduced tumor growth and prolonged survival. The results represent the first demonstration of PNA-targeting a lncRNA in a solid malignancy *in vivo* and suggest a novel cancer therapeutic approach for ovarian, breast and other solid cancers.

## Results

### Inhibiting HOTAIR and EZH2 alters platinum sensitivity and cell behaviors

**Figure 1:**
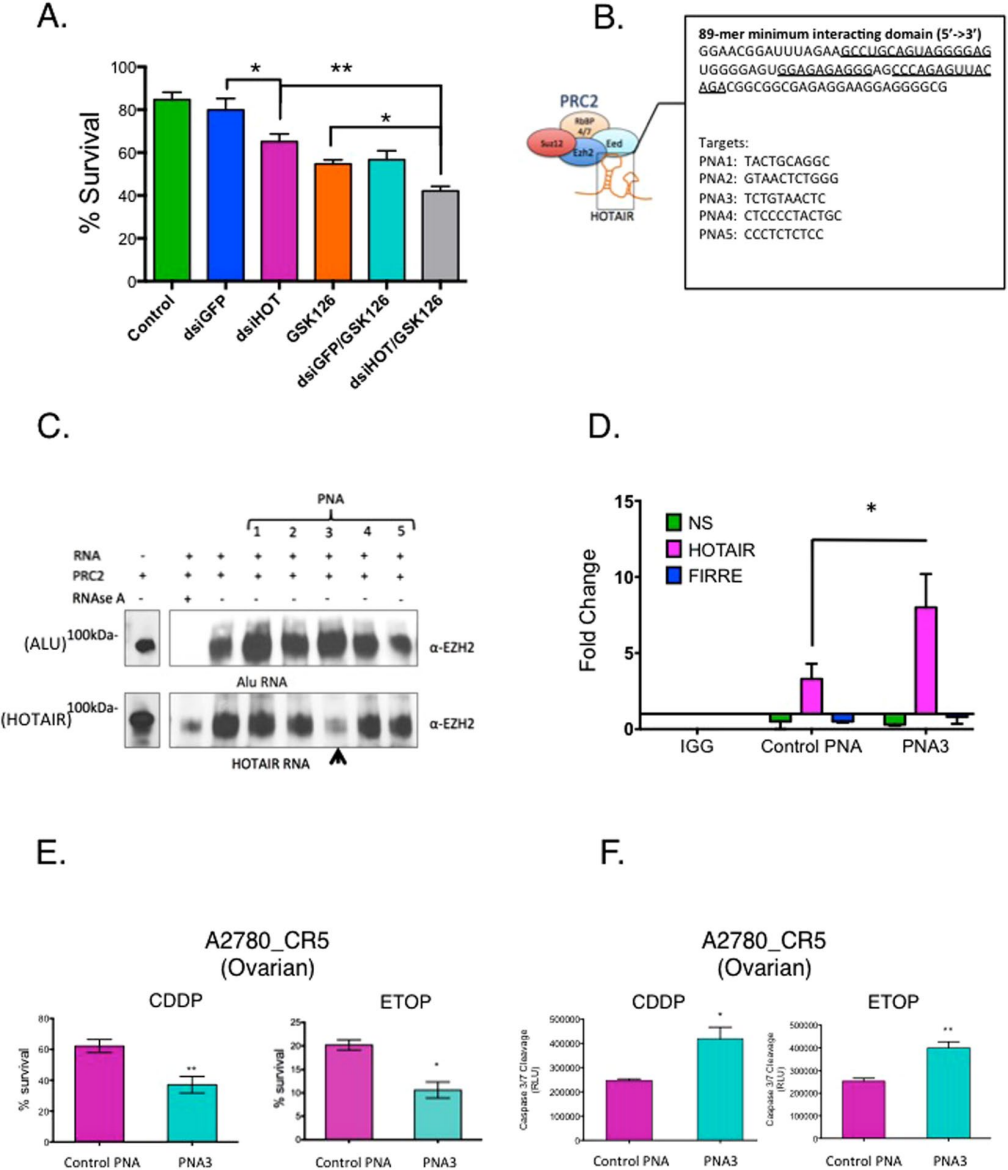
Inhibition of HOTAIR and EZH2. **(A)** A2780_CR5 cells were treated with EZH2 inhibitor (GSK126 5 μM) or transfected with either dsiGFP or dsiHOTAIR (50 nM) for 24 hrs trypsinized and replated into 96 well plates in triplicate for 72 hrs. Cell survival was determined with MTT assay. **(B)** 89-mer minimum-interacting region of HOTAIR and the sequence of the PNAs used in this study. **(C)** *In vitro* transcribed and biotinylated HOTAIR or ALU RNA (1 μM) were incubated with PNA1-5 (1 μM) for 1 hr at 25 °C followed by pull-down with streptavidin coated protein A/G plus agarose beads. 50 ng of recombinant EZH2 was added and binding was observed in a polyacrylamide gel. **(D)** PNA3 or control PNA were biotinylated and incubated with MCF-7 cell lysate for 1 hr followed by a modified ChIRP assay. Graph represents the fold change of HOTAIR as measured by qRT-PCR compared to control non-specific gene and lncRNA FIRRE. **(E)** A2780_CR5 cells were treated with either PNA3 or control PNA (1 μM) for 24 hrs, and re-plated for clonogenic survival and treated with either CDDP (30 μM) or etoposide (5 μM) for 3 hrs and 24 hrs post-treatment **(F)** Caspase 3/7 cleavage assay was performed. All western blot data were cropped and acquired under same experimental conditions. Asterisks indicate *P* < 0.05 (*) or *P* < 0.01 (**).

Inhibiting either HOTAIR^9^ or EZH2^19, 20^ has been reported to reduce tumorigenesis and increase survival *in vivo*. To examine the effect of inhibiting both HOTAIR and EZH2, we treated a highly platinum-resistant ovarian cancer cell line (A2780_CR5) with dsiRNA targeting HOTAIR and (or) a pharmacological inhibitor of EZH2 (GSK126) and performed survival assays and observed an additive (*P* < 0.05) on drug sensitivity and survival (Fig. 1A, inhibiting both HOTAIR and EZH2 vs. inhibiting either factor alone). To disrupt the HOTAIR-EZH2 interaction, we targeted the 89-mer minimum interacting region of HOTAIR, which has been recently reported to bind EZH2^16^. By using mFold^21^ to validate the predicted secondary structure of this site, we observed a highly predicted, single-stranded region complementing previous such structures (Supplementary Fig. S1A; total of 19 predicted structures) and designed peptide nucleic acids (PNAs) complementary to the single stranded region of the 89-mer domain (Fig. 1B, red lines). Individual PNAs (PNAs 1–5, Supplementary Table S1) were combined with *in vitro* transcribed, biotinylated full-length HOTAIR (1μM) and recombinant EZH2 (Supplementary Fig. S1B). Of the five PNAs examined (1 μM each), only PNA3 reduced (approximately 80%) the HOTAIR-EZH2 interaction (Fig.1C and Supplementary Fig. S1C). As such, PNA4 was used as the control PNA, as it had no effect on HOTAIR-EZH2 interaction. No effect of the other PNAs was observed and importantly none of the PNAs altered the EZH2-ALU (control RNA) interaction (Fig. 1C and Supplementary Fig. S1D), further demonstrating PNA3-specific inhibition of the HOTAIR-EZH2 interaction. The ability of PNA3 to bind *in vitro* transcribed HOTAIR using gel shift assay. At 1 × 1^−2^ μM PNA3, a shifter band was observed (Supplementary Fig. S1E), whereas no observable band shift was seen with control PNA. PNA3 bound HOTAIR from HEK293 cell lysate ectopically overexpressing full length HOTAIR (Fig. 1D; 8-fold enrichment of HOTAIR with PNA3 compared to control PNA, determined by qRT-PCR), whereas a no such enrichment was observed using non-specific primer control and primers corresponding to the lncRNA FIRRE. In order to exclude possible off-targets, the sequences of each PNA was aligned to the genome. No significant genes were found to interfere specifically with PNA3, whereas several genes were found to be complementary to PNA5 (Supplementary Table S2).

To examine the effect of PNA3 on ovarian and breast cancer cell lines overexpressing HOTAIR (Supplementary Fig. S1F, Supplementary Table S3), cells were treated with PNA3 or control PNA (1μM each) alone or in combination with chemotherapeutics cisplatin (CDDP) or etopiside, functional assays were performed (clonogenic survival, caspase 3/7 cleavage, and proliferation assays), and levels of EZH2 and histone H3 lysine K27 trimethylation (H3K27me3) were examined. PNA3-CDDP or -etoposide treatment inhibited (*P* < 0.05) clonogenic survival of CDDP-resistant A2780_CR5 (0.8-fold or 0.5-fold respectively, compared to control PNA-chemotherapy combinations; Fig. 1E), Kuramochi (lesser extent: 0.9-fold CDDP, 0.9-fold etoposide; Supplementary Fig. S1G), MCF-7 (0.4-fold CDDP, 0.8-fold etoposide) and MDA-MB-231 (0.7-fold CDDP, 0.3-fold etoposide) (Supplementary Fig. S2A,B). The combinations had no effect on survival of CDDP-sensitive A2780p cells (Supplementary Fig. S2C). SKBR-3 breast cancer cells do not form colonies and we were unable to generate clonogenic survival data. Consistent with the clonogenic data, increased (*P* < 0.05) caspase 3/7 cleavage was observed for ovarian (Fig. 1F, Supplementary Fig. S2D; but not A2780p, Supplementary Fig. S2C) and breast (Supplementary Fig. S2F-H) cancer cell lines. No effect of PNA3 on cell proliferation (Supplementary Fig. S3A-F) was observed and no significant change (<5%) in HOTAIR expression was observed in cell lines treated with PNA (data not shown).

**Figure 2:**
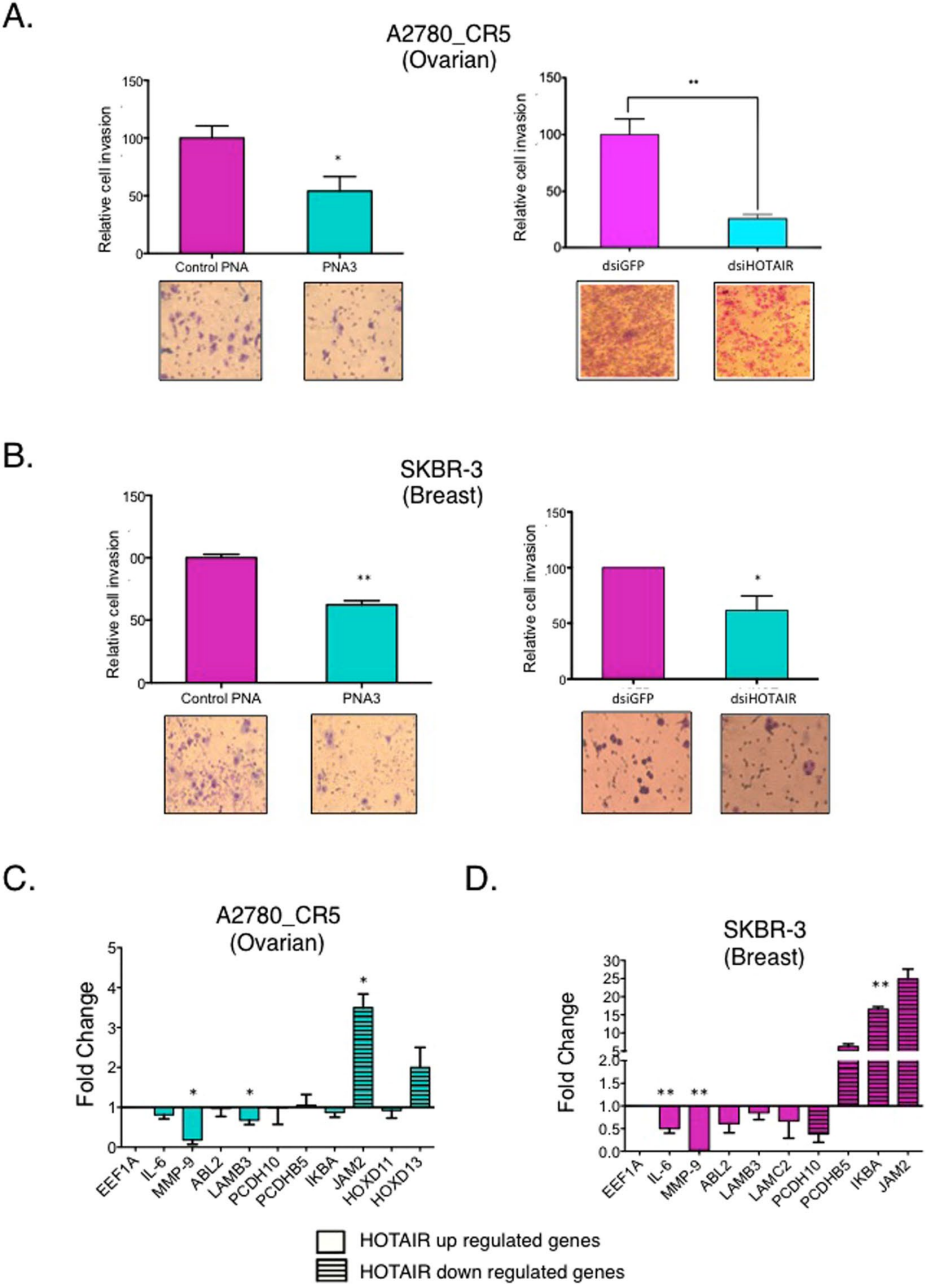
Combination treatment with CDDP and PNA3 reduces cell invasion, and HOTAIR target gene expression in breast and ovarian cancer cells. (**A**) A2780_CR5 or (**B**) SKBR-3 cells were treated with either PNA3 or control PNA (1 μM) or transfected with either dsiGFP or dsiHOTAIR for 24 hrs and invasion assays were performed. Representative images of invading cells were counted under a light microscope (20X magnification) and normalized to control (**C**) A2780_CR5 and (**D**) SKBR3 cells were treated with either PNA3 or control PNA (1 μM) for 48 hrs and qPCR was performed for HOTAIR targets: IL-6, MMP-9, ABL-2, LAMB3, LAMC2, PCDH10, PCDHB5, IKBA, JAM2, and HOXD13. Genes were omitted from certain cell lines due to undetectable endogenous expression. Asterisks indicate *P* < 0.05 (*) or *P* < 0.01 (**).

Overexpression of HOTAIR and metastasis of ovarian, breast and lung cancers has been reported^22^, and we and others demonstrated that HOTAIR induced MMP-9 expression and increased cancer cell invasion^9^. Treatment of ovarian and breast cancer cells with PNA3 decreased (*P* < 0.05) invasion of A2780_CR5 (2-fold, Fig. 2B), Kuramochi (0.6-fold, Supplementary Fig. S3G) and SKBR-3 (2-fold, Fig. 2B). The effect of PNA3 on invasion was similar to siRNA knock-down of HOTAIR expression in these cell lines (Fig. 2A,B). However, A2780p and MDA-MB-231 invasion was not decreased by PNA3 treatment (Supplementary Fig. S3H,I).

### PNA3 inhibits HOTAIR-induced target gene expression and NF-κB activity

To investigate the effect of PNA3 on HOTAIR target gene expression, we measured levels of genes previously shown to be up- (*IL6, MMP9, ABL2, LAMB3, LAMC2*) or down- (*IKBα, JAM2, HOXD11, HOXD13, PCDH10, and PCDHB5*) regulated by HOTAIR^5^ using qRT-PCR (48 hrs post-PNA treatment). When compared to control PNA, we observed consistent decreased expression of *IL-6* and *MMP-9* and increased *JAM2, HOXD13* and *IKBA* expression in the majority of ovarian (Fig. 2C, Supplementary Fig. S4A,B) and breast (Fig. 2D, Supplementary Fig. S4C,D) cancer cell lines examined (some genes omitted from graphs due to very high threshold cycle (CT) values or no detectable expression in some cell lines examined). Interestingly, although PNA3 treatment altered HOTAIR target gene expression, no effect on total EZH2 and H3K27me3 levels (Supplementary Fig. S4E) was observed.

**Figure 3:**
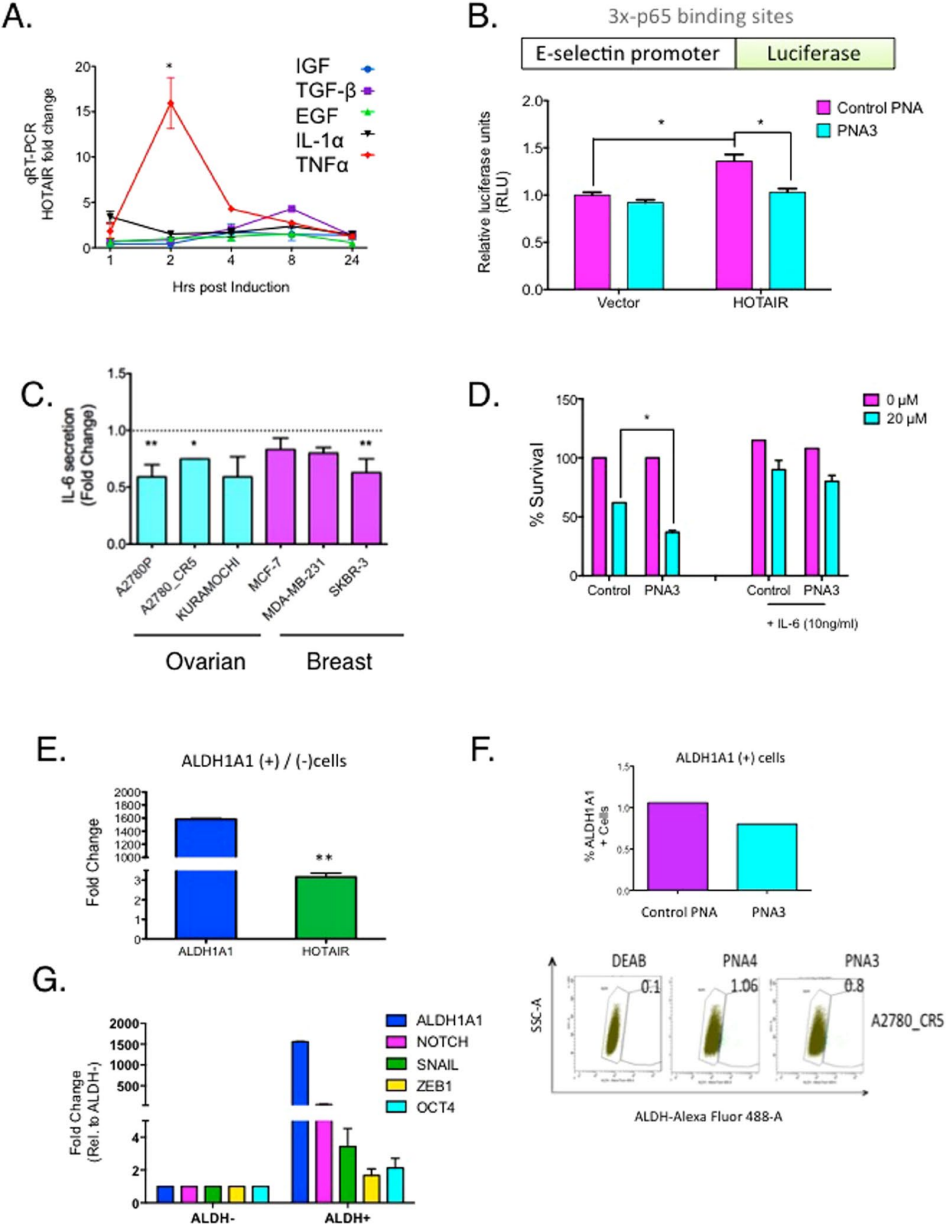
PNA treatment reduces NF-κB transcriptional activity and IL-6 secretion. **(A)** A2780p cells were treated with IGF, TGF-β, EGF, IL-1α, and TNF-α (10 ng/mL) and qPCR was performed to measure HOTAIR. **(B)** Luciferase activity of A2780p cells transfected with E-Selectin promoter carrying 3x p65-NF-κB binding sites and either ectopically overexpressing HOTAIR or vector control treated with or without PNA3 or control PNA. **(C)** IL-6 secretion by ovarian (A2780p, A2780_CR5, Kuramochi) and breast (MCF-7, MDA-MB-231 and SKBR-3) cancer cells: 48 hrs post-treatment with either PNA3 or control PNA (1 μM). **(D)** Survival of A2780P cells treated with CDDP (20μM) using conditioned media from PNA3- or control PNA-treated A2780_CR5**. (E)** ALDH1A1 activity was determined using flow cytometry to separate ALDH1A1(+) and ALDH(−) cells from platinum-resistant (A2780_CR5) ovarian cancer cells. RNA was isolated from the two different cell populations and qRT-PCR for expression of ALDH1A1 (blue bar) HOTAIR (green bar) was performed. HOTAIR expression in ALDH(+) was normalized to ALDH(−) cells. **(F)** A2780_CR5 cells were treated with PNA3 (1 μM) or control PNA (1 μM) for 24 hrs. ALDH1A1 activity was determined by flow cytometry and total ALDH1A1(+) cells were compared to ALDH1A1(−) cells. **(G)** ALDH1A1 activity was determined using flow cytometry to separate ALDH1A1(+) and ALDH(−) cells from platinum-resistant (A2780_CR5) ovarian cancer cells. RNA was isolated from the two different cell populations and qRT-PCR for expression of stemcell markers: NOTCH1, SNAIL, ZEB1, and OCT4 was performed in ALDH(+) and normalized to ALDH(−) cells. Asterisks indicate *P* < 0.05 (*) or *P* < 0.01 (**).

A positive association between HOTAIR and the master transcription factor NF-κB has been reported (Chu *et al*., 2011), and we demonstrated NF-κB-mediated transcriptional regulation of HOTAIR induced epigenetic silencing of Iκ-Bα, resulting in a positive feedback loop that ultimately increased NF-κB activation in ovarian cancer^9, 23^. We thus performed a cytokine/chemokine screen and then measured HOTAIR levels. Of the cytokines examined, HOTAIR expression was increased (*P* < 0.05) by TNF-α (>15-fold) and TGF-β (5-fold) compared to control (Fig. 3A), in agreement with previous findings^9, 23^. To confirm HOTAIR induction of NF-κB, we used a previously reported luciferase reporter construct containing the E-selectin promoter (861 base pairs containing 3 canonical NF-κB-p65-binding sites as a positive control; Fig. 3B)^24^. Cells were transfected with luciferase constructs and treated with control PNA or PNA3 for 24 hrs and luciferase activity was measured. We observed a 1.4-fold increase (*P* < 0.05) in luciferase activity by ectopic overexpression of HOTAIR compared to vector control (Fig. 3B), which was decreased (*P* < 0.05) by PNA3 (Fig. 3B). Furthermore, PNA3 treatment of ovarian and breast cancer cells decreased (*P* < 0.05) IL-6 secretion into the media (Fig. 3C). Because secreted IL-6 contributes to chemoresistant cancer stem cells by inducing aldehyde dehydrogenase (ALDH1A1) we performed a survival assay with conditioned media (CM) from ovarian cancer cells treated for 24 hours with either PNA3 or control PNA. Increased (*P* < 0.05) sensitivity to CDDP for cells treated with PNA3 CM *vs*. control CM was observed, and this effect was rescued with recombinant IL-6,, suggesting that inhibiting IL-6 secretion is a contributing factor to chemosensitivity (Fig. 3D), an effect further supporting claims from our previous work^9^. Furthermore, as the IL6-STAT3 axis regulates ALDH1A1 activity and contributes to ovarian cancer stem cell enrichment^25, 26^, we measured HOTAIR levels in ALDH1A1 positive A2780_CR5 vs. negative cells. An approximate 1600-fold increase in ALDH1A1 expression (Fig. 3E) was detected as well as a 3-fold increase in HOTAIR expression in ALDH1A1 positive ovarian cancer cells relative to negative cells (Fig. 3E). Treatment of A2780_CR5 cells with PNA3 decreased (25%) ALDH1A1 activity (Fig. 3F), suggesting HOTAIR inhibition with PNA could reduce the cancer stem cell population.

### Effect of pHLIP-conjugated PNA3 on CDDP sensitivity, tumor formation and survival

**Figure 4:**
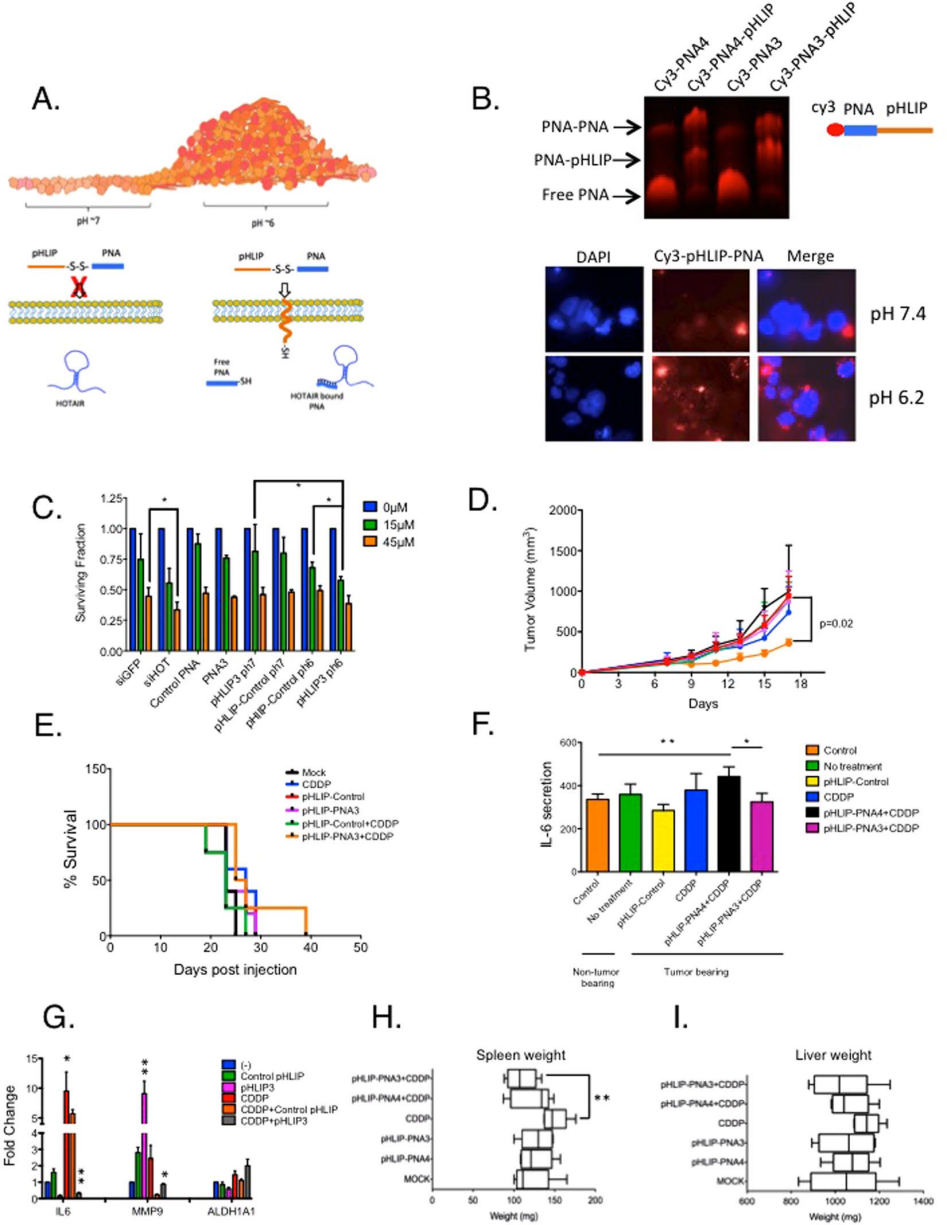
pHLIP-PNA peptide is pH specific and can resensitize ovarian cancer cells to cisplatin *in vivo* and increases survival. (**A**) Diagram illustrating the mechanism for pHLIP-PNA mediated targeting of lncRNA HOTAIR. (**B**) pHLIP-peptide conjugation to cy3 labeled PNA in a 4% TBE-acrylamide gel and immunofluorescence showing pHLIP-PNA entering cells in low and high pH conditions (pH 6.2 vs pH 7.4). (**C**) A2780_CR5 treated with pHLIP-PNA3 or pHLIP-PNA-Control (1 μM) for 1 hr followed by a 3 hr treatment with indicated concentrations of CDDP (0, 15, 45 μM). Survival was measured with MTT (72 hrs post treatment). (**D**) Tumor growth in response to PNA or cisplatin (CDDP) treatment; arrows represent 1 mg kg^−1^ PNA and/or 2 mg kg-1 CDDP *n* = 5. (**E**) Survival in response to anti-lncRNA treatment. Cutoff criteria included tumor volume greater than 1 cm^3^ or protocol-mandated euthanasia; *n* = 5 for all groups. (**F**) Blood IL-6 levels at the last biweekly collection; n = 5 for all groups. (**G**) Gene expression analysis as measured by qRT-PCR for HOTAIR target genes: IL-6, MMP-9, and ALDH1A1. (**H**) Average spleen weight for all groups of mice collected at the time of euthanasia; n = 5 mice per group. (**I**) Average liver weight for all groups of mice collected at the time of euthanasia; n = 5 mice per group. Asterisks indicate *P* < 0.05 (*) or *P* < 0.01 (**).

An acidic tumor micro-environment (pH~6 vs pH 7), due to increased glycolysis resulting in lactic acidosis (Warburg effect;^27^), has been reported for solid tumors including breast^28^ and ovarian^29^ cancers. To target PNAs to the acidic tumor microenvironment, we used pH-low insertion peptides (pHLIPs), which are unstructured peptides in either neutral pH or basic pH and can thus interact with the outer surface of lipids in a reversible manner (Fig. 4A). Based on a previous report of successful pHLIP-PNA targeting a non-coding RNA (microRNA-155)^14^, we conjugated thiolated pHLIP peptide to PNA3 and control PNA (verified using tricine SDS-PAGE gel, Fig. 4B) and examined pHLIP-PNA A2780_CR5 cell entry (normal pH 7.2 vs. acidic pH 6.0 conditions) using immunofluorescence. Signals in cytoplasm, nucleus and cell periphery were observed (Fig. 4B). To examine PNA3 resensitization of A2780_CR5 cells to CDDP, cells were treated with either pHLIP-conjugated-PNA3 or -control PNA under normal or acidic pH and various CDDP concentrations (0, 15, 45 μM) and an MTT survival assay was performed. No change in cell survival was observed between pHLIP-PNA3 and pHLIP-control under normal pH; however in pH 6, pHLIP-PNA3 decreased (*P* < 0.05) survival (Fig. 4C), indicating HOTAIR targeting and altered CDDP-sensitivity under acidic conditions. To investigate anti-tumor properties of pHLIP-PNA3 *in vivo*, BALB/c-nu/nu mice were injected with CDDP-resistant A2780_CR5 (2 × 10^6^ cells subcutaneously). Once tumors reached ~200 mm^3^, mice were injected intravenously (biweekly for 2 weeks) with pHLIP-PNA3 (1 mg/kg), pHLIP-control PNA (1 mg/kg) and/or CDDP (2 mg/kg i.p.) (Supplementary Fig. S4F). Tumor volume was reduced (*P* = 0.02) in mice co-administered pHLIP-PNA3 + CDDP compared to pHLIP-control PNA + CDDP (Fig. 4D), and tumor volume in mice treated with either pHLIP-PNA alone was similar to vehicle-treated mice (Fig. 4D). Survival of mice treated with pHLIP-PNA3 was increased (64% vs. mock; Fig. 4E). Body weight among groups was similar (Supplementary Fig. S4G), demonstrating that PNAs are non-toxic *in vivo*.

### pHLIP-PNA3-cisplatin combination treatment decreases HOTAIR targets *in vivo*

As a positive correlation between tumor growth and the pro-inflammatory cytokine IL- 6 has been described^30^ (Fig. 3C), and we recently demonstrated that HOTAIR upregulated both IL-6 and MMP-9 in ovarian cancer cells^9^, it was of interest to examine the effect of PNA3 on IL-6 *in vivo*. In ovarian tumor-bearing mice, blood IL-6 levels increased (*P* < 0.01) after treatment with CDDP alone or pHLIP-control PNA + CDDP, and IL-6 blood levels were reduced (*P* < 0.05) after pHLIP-PNA3 + CDDP treatment compared to pHLIP-PNA control + CDDP (Fig. 4F). In addition, tumor expression of IL-6, MMP-9 and ALDH1A1 was decreased (*P* < 0.05) in mice treated with pHLIP-PNA3 compared to control (Fig. 4G). The increase in IL-6 levels with pHLIP-PNA4 could be due to the activation of pro-inflammatory pathways in the tumors generated by the penetration of pHLIP-PNA4. Taken together, the results demonstrate that pHLIP-PNA3-mediated HOTAIR inhibition reduces ovarian tumor levels of IL-6, MMP-9, and ALDH1A1, increases CDDP sensitivity and improves overall survival.

Platinum-based therapies have been reported to affect the liver and spleen^31^. CDDP treatment increased (*P* < 0.01) spleen size compared to mock treated (Fig. 4H), but interestingly the combination of pHLIP-PNA3 with CDDP abrogated (*P* < 0.01) the CDDP-induced increase in spleen size (Fig. 4H). A similar trend for liver size was observed (Fig. 4I) albeit not statistically significant. Furthermore, no apparent histological changes (based on H&E staining) were seen (Supplementary Fig. S4H), but H&E slides of ovarian tumors from CDDP-treated mice exhibited fewer cells (Supplementary Fig. S4H), indicating cell death.

## Discussion

The lncRNA HOTAIR is frequently overexpressed in solid tumors^32^ and correlates with disease progression chemoresistance and poor patient prognosis^9, 33^. The oncogenic activity of HOTAIR is dependent upon its interaction with the PRC2 complex, specifically EZH2, an epigenetic modifier frequently perturbed in cancer^34^. In the current study, by inhibiting the activity of the EZH2-binding partner HOTAIR, which is frequently co-expressed in EZH2-overexpressing cancers^35^, we demonstrate a novel and effective strategy for resensitizing resistant ovarian tumors to platinum. We describe a PNA-based approach to block the ability of HOTAIR to interact with EZH2 and subsequently inhibit HOTAIR-EZH2 activity *in vitro* and *in vivo*.

To initially examine the impact of combined inhibition of HOTAIR and EZH2, we treated ovarian and breast cancer cells with HOTAIR siRNA plus EZH2 catalytic activity inhibitor (GSK126), observing increased chemotherapy sensitivity and reduced cell survival (Fig. 1A). Furthermore, similar to combining genetic knockdown with pharmacologic inhibition, using a PNA to block to the recently described EZH2-interactiing domain of HOTAIR^16^ and disrupt the HOTAIR-EZH2 interaction resensitizes cancer cells to clinically relevant cytotoxic chemotherapies (Figs 1E,F, 2, 3 and 4), reduces cell invasion (Fig. 2A,B) and decreases NF-κB transcriptional activity (Fig. 3B) and IL-6 and MMP-9 expression *in vivo* (Fig. 4F,G). Similar efficacy of PNA in cell lines with low endogenous HOTAIR such as SKBR-3 and MDA-MB-231 cells could be due to a “dosage effect” where lower HOTAIR levels are more efficiently inhibited by PNA. Our findings on IL-6, a pro-survival cytokine that can transform cells to a “pro-inflammatory cell”, also indicate a potential approach for inhibiting IL-6 secretion, tumor progression and chemotherapy resistance development.

Approaches for targeting non-coding RNAs in tumors *in vivo* include siRNA-mediated knockdown and locked nucleic acids. We show that PNA-pHLIP conjugation is effective in an acidic tumor microenvironment, suggesting that the approach could overcome the impact of Warburg effect, a well-known fundamental aspect of malignant transformation^27^. We further demonstrate that PNA-pHLIP can be safely (based on no change in body weight) and effectively (based on reduced tumor burden) combined with cytotoxic chemotherapy, including platinum-based drugs currently used in the clinic. Importantly, PNA3-pHLIP treatment lowers both tumor and blood levels of IL-6, suggesting that impacting the local (tumor) microenvironment may result in systemic (peripheral) effects, such as reducing inflammation. Although the observed improvement in mouse survival may be considered modest (2 weeks compared to control), the duration represents nearly a two-year increase when converted into human years.

Solid tumors are characteristically associated with an acidic environment as well as reduced oxygen levels, which activates HIF-1α^28^, an oncogene that further promotes tumor growth under low oxygen levels and increase cancer stem cells population^36^. Interestingly, HIF1α was recently shown to regulate HOTAIR expression under hypoxic conditions^37^. As IL-6-STAT3 axis can induce expression of ALDH1A1, a cancer initiating cell marker^38^, it was of interest to determine the effect of PNA treatment on this cell population. We show ALDH1A1(+) ovarian cancer cells display increased HOTAIR expression (Fig. 3E) and PNA3 treatment decreases ALDH1A1 level *in vitro* (Fig. 3F) and *in vivo* (Fig. 4G). The observation that PNA3 treatment also decreased IL-6 levels (Figs 3C and 4F,G) indicates that PNA3-targeting enhanced the response of ovarian cancer initiating cells to CDDP (Figs 3E,F and 4G). Our future work will investigate the role of HOTAIR and the lncRNA as a possible therapeutic target in the ovarian cancer stem cell population.

In conclusion, we report for the first time an anti-lncRNA targeting approach in ovarian tumors *in vivo*. The findings warrant further development of this strategy for targeting oncogenic lncRNAs as a therapeutic strategy in solid tumors.

### Experimental Procedures

Detailed experimental procedures can be found in extended experimental procedures. All methods were performed in accordance with the relevant guidelines and regulations approved by Indiana University.

### Cell lines, culture conditions and reagents

Epithelial ovarian cancer cell lines (KURAMOCHI, A2780p, A2780_CR5; Supplementary Table S3) were maintained in RPMI 1640 medium. Cisplatin-resistant A2780_CR5 was derived from A2780p (parental) by continuous exposure to cisplatin^39^. Breast cancer cell lines (MCF-7, MDA-MB-231 and SKBR3; Supplementary Table S3) were maintained in EMEM or McCoys media (Invitrogen, Carlsbad, CA). Cell lines were authenticated in 2012 by ATCC and tested for mycoplasma contamination (Manassas, VA). Cisplatin (CDDP) was purchased from Calbiochem (Billerica, MA), and etoposide was purchased from Santa Cruz Biotech (Santa Cruz, CA). LZRS-HOTAIR was a gift from Dr. Howard Chang (Stanford University; Addgene plasmid #26110). Full-length HOTAIR was cloned into pAV5S vector containing a 98-mer aptamer sequence and as a vector control, aptamer cloned into pAV5S was used to account for any possible RNA-dependent signaling effects^40^.

### Proliferation MTT assays

Cells were grown in 6 cm culture plates until 70% confluence and treated with either PNA3 or control PNA (1 μM final) for 24 hrs. Next day, plates were trypsinized, counted, 2 × 10^3^ cells were seeded into 96-well and MTT assay was performed as previously described^9^.

### Cell invasion assays

Cells were grown in 6 cm culture plates until 70% confluence and treated with either PNA3 or control PNA (1 μM final) for 24 hr. Next day, 50,000 cells were seeded inside a matrigel invasion chamber insert (Corning Inc., Corning, NY) in serum free media supplemented with 0.1% BSA. Cells were fixed 48hrs later and analyzed^9^.

### *In vitro* transcription RNA

Full length T7-promoter driven HOTAIR and ALU cDNAs were cloned into pcDNA3.1 with a single NHEI restriction site after the transcription stop site. DNA was linearized with NHEI and DNA was *in vitro* transcribed into RNA according to manufactures protocol (New England Biolabs, Ipswich, MA). The total RNA was purified and DNAseI treated and purified per manufacturers protocol (Qiagen).

### Biotinylation, folding, and immunoprecipitation of RNA

Purified RNA (1.67 μM) was 3′-biotinylated according to manufacturers protocol (Thermo Scientific). After biotinylation, RNA was purified and folded. Folded 3′ biotinylated ALU or HOTAIR RNA was incubated with individual peptide nucleic acids (PNAs) (5 μM final) (Supplementary Table S1) PNA Bio (Thousand Oaks, CA) in 10 μL of 1x folding buffer supplemented with RNAse inhibitor (Thermo Scientific) and bovine serum albumin (5 μg BSA) for 30 min at 37 °C. Next, streptavidin HRP antibody (Cell Signaling, Danvers, MA) (Supplementary Table S4) was added to binding buffer supplemented with RNAse inhibitor and incubated at 4 °C for 1 hr. Next, protein A/G plus agarose beads (25 μL; Santa Cruz Biotech) were added and placed into 4 °C rotator for 1 hr. The beads were washed and recombinant polycomb repressive complex 2 (PRC2, 0.1 nM final, Active Motif, cat #31387) was added and incubated for 3hr at 4 °C on a rotator. After incubation beads were washed 3x with 1X IP buffer supplemented with RNAsein and run on BioRad precast polyacrylamide gel.

### Synthesis of PNAs and pHLIP-antiLinc constructs

The PNAs were purchased (PNA Bio) containing cell-penetrating peptide (CPP) RRRQRRKKR. The pHLIP peptide was purchased from (New England Peptide, Gardner, MA): AAEQNPIYWARYADWLFTTPLLLLDLALLVDADEGT(CNPys)G. pHLIP-antiLinc constructs, were conjugated to the C-terminus of thiolated PNA using a cysteine group derivatized with 3-nitro-2-pyridinesulphenyl (NPys) similar to the recently published report^14^. To synthesize pHLIP-antimiR constructs, pHLIP-Cys(NPys) and antiLinc PNA (peptide:PNA 1:1.3) were reacted overnight in the dark in a mixture of DMSO/DMF/0.1 mM KH_2_PO_4_ pH 4.5 (v/v 3:1:1). The thiolated PNAs used in the study are listed in Supplemental Table S1.

### Clonogenic survival and Caspase 3/7 cleavage assays

24 hrs after treatment with PNA, cells were washed with 1X PBS and were either not treated or treated with indicated concentrations of CDDP or etoposide. Cleaved Caspase 3/7 activity, indicative of apoptosis, was detected according to manufacturers protocol (Promega, Madison, WI). Percent survival of treated cells was calculated relative to untreated samples.

### Aldefluor assay and flow cytometry

ALDH1 enzymatic activity was measured using the Aldefluor assay kit (Stemcell Technologies, Vancouver, Canada) following the manufacturer’s instructions and as we have described^41^.

### ChIPNA assay

Cells were treated with 1 μM of biotinylated PNA3 or control PNA was added. 24 hrs later cells were, trypsinized, and fixed with 4% formalin. The nuclei were isolated as previously described^9^ then resuspended in nuclei ChIP lysis buffer The soluble fraction was incubated with anti-streptavidin antibody (Supplementary Table S4) for 2 hrs followed by binding of protein A/G plus agarose beads (Santa Cruz Biotech) for an additional 2 hrs at 4 °C. Beads were washed 3 times with wash buffer at 4 °C and then Proteinase K treated. Nucleic acid was separated with TRIzol and RNA was purified using RNAeasy column (Qiagen). RNA isolate (1 μL) was used per well for qRT-PCR analysis to confirm lncRNA retrieval. LncRNA FIRRE was used as a negative control, LncRNA ANRIL was used as a positive control.

### Mouse xenograft experiments

All animal studies adhered to ethical regulations and protocols approved by the Institutional Animal Care and Use Committee of Indiana University. To assess tumorigenicity of cells, cultured A2780_CR5 cells were re-suspended in 1:1 PBS/matrigel (BD Biosciences) and 2 × 10^6^ cells were injected subcutaneously into the left flank of 3- to 4- week-old female nude athymic mice (BALB/c-nu/nu; Harlan, Indianapolis, IN), as described^41, 42^. Engrafted mice (n = 6 per group) were inspected three times per week for tumor appearance by visual observation and palpation. Once tumors were ~200 mm^3^, mice were treated with either CDDP (2 mg kg^−1^) or PNA (1 mg kg^−1^) or both CDDP and PNA biweekly for two weeks. Blood samples were collected by puncturing the left lateral saphenous vein with a needle and collected using a capillary tube. Tumor length (l) and width (w) were measured biweekly using digital calipers and tumor volume (v) was calculated as v = ½ × l × w^2^. The investigator measuring tumor size was blinded to the treatment groups. Mice were sacrificed when tumor diameter reached 2 cm or at the end of study.

### ELISA and cytokine release assays

Twenty-four hours post-PNA treatment, cells were rinsed with 1X PBS and incubated in serum-free RPMI medium. Total cell counts were determined and ELISA was performed using kits and procedures from R&D systems (Minneapolis, MN; Cytokine release assay,) and eBiosciences (San Diego, CA; IL-6 ELISA).

### Luciferase assays

Cells were seeded in 96-well plates (10^4^ cells/well) and transfected with pGL3-E-selectin vector (300 ng construct/transfection). Transfection efficiency was normalized with co-transfected PGL4 Renilla plasmid (100 ng). Twenty-four hours after transfection, cells were treated with PNA3 or Control PNA (1μM) for indicated times. Luciferase activity was analyzed using the Dual Luciferase Reporter Assay System (Promega) and a Thermo Scientific Multilabel Plate Reader.

### RNAi

The dsiRNA sequences used targeting human HOTAIR (Sense strand 5′-UUCUAAAUCCGUUCCAUUCCACUGCGA-3′, and antisense strand 5′-/5Phos/GCAGUGGAAUGGAACGGAUUUAGAA-3′) or negative control RNA targeting GFP (Sense strand 5′-CUACAACAGCCACAACGUC-3′, and antisense strand 5′-/5Phos/GACGUUGUGGCUGUUGUAG-3′). DsiRNAs were transfected into cells using Lipofectamine 2000 (Invitrogen; per manufacturer protocol).

### Immunoblot analysis

Cells were lysed in RIPA lysis buffer and protein (approximately 5–10 μg) was loaded on precast 7.5% TGX gels (BioRad, Hercules, CA), blotting was performed as described previously^43^ and antibodies for EZH2, H3, H3K27me3, Streptavidin, and Beta-Tubulin (Supplementary Table S4).

### RNA extraction and quantitative RT-PCR (qPCR)

RNA was extracted from cell lines and tumors and using RNeasy kit (Qiagen, Venlo, Limburg), cDNA was prepared using MMLV RT system (Promega), and qPCR) was performed with total cDNA and primers for indicated genes and GAPDH or EEF1A as the endogenous control (Supplementary Table S5), using Applied Biosystems 7500 Fast RT-PCR system (Life Technologies, Grand Island, NY) and corresponding software, as we have described^41^. Primers used for can be found in Supplementary Figure S4.

### Immunofluorescence quantification

A2780_CR5 cells were plated on glass slides (50,000 cells/well) and incubated at 37 °C, and 24 hrs later incubated with 100 nM cy3-pHLIP-PNA3 or cy3-pHLIP-Control PNA for 1 hr at 37 °C, washed 3x with 1x PBS. Slides were then prepared as described previously^9^.

### Statistical analysis

All data are presented as mean values ± SD of at least three biological experiments unless otherwise indicated. IC_50_ values were determined by Prism 6 (GraphPad Software, San Diego, CA), using logarithm normalized sigmoidal dose curve fitting. The estimate variation within each group was similar therefore student’s *t*-test was used to statistically analyze the significant difference among different groups by using Prism 4.0 (GraphPad Software). For mouse xenograft study, statistical significance was determined using student two-tailed t-test.

## Electronic supplementary material

Supplementary Info
