# Supplementary material for: Therapeutic targeting using tumor specific peptides inhibits long non-coding RNA HOTAIR activity in ovarian and breast cancer
Source: Sci Rep. 2017 Apr 18;7:894. doi: 10.1038/s41598-017-00966-3 (PMC5429858; doi:10.1038/s41598-017-00966-3)
Supplement: Supplementary file 1 — Supplementary Info [file 41598_2017_966_MOESM1_ESM.pdf]

Therapeutic targeting using tumor specific peptides inhibits long non-coding RNA HOTAIR activity in ovarian cancer

Ali R. Özeş[1], Yinu Wang[2], Xingyue Zong[2], Fang Fang[2], Jay Pilrose[2], and Kenneth P. Nephew[1,2,3,4,5*]

Authors' affiliations:

[1]Molecular and Cellular Biochemistry Department, Indiana University, Bloomington, IN 47405 USA

[2]Medical Sciences Program, Indiana University School of Medicine, Bloomington, IN 47405 USA

[3]Indiana University Melvin and Bren Simon Cancer Center, Indianapolis, Indiana 46202 USA

[4]Department of Obstetrics and Gynecology, Indiana University School of Medicine, Indianapolis, IN, 46202 USA

[5]Department of Cellular and Integrative Physiology, Indiana University School of Medicine, Indianapolis, IN 46202, USA

***Corresponding Author:** Kenneth P. Nephew, Ph.D.
Professor
Indiana University School of Medicine
Jordan Hall 302
1001 E. Third Street
Bloomington, IN 47405-4401
Email: knephew@indiana.edu
Phone: (812) 855-9445

Extended experimental procedures

Material and Methods

Cell lines, culture conditions and reagents. Epithelial ovarian cancer cell lines (KURAMOCHI, A2780p, A2780_CR5; Supplementary Table S1) were maintained in RPMI 1640 medium. Cisplatin-resistant A2780_CR5 was derived from A2780p (parental) by continuous exposure to cisplatin (Li et al., 2009). Breast cancer cell lines (MCF-7, MDA-MB-231 and SKBR3; Supplementary Table S1) were maintained in EMEM or McCoys media (Invitrogen, Carlsbad, CA). Cell lines were authenticated in 2012 by ATCC and tested for mycoplasma contamination (Manassas, VA). Cisplatin (CDDP) was purchased from Calbiochem (Billerica, MA), and etoposide was purchased from Santa Cruz Biotech (Santa Cruz, CA). LZRS-HOTAIR was a gift from Dr. Howard Chang (Stanford University; Addgene plasmid #26110). Full-length HOTAIR was cloned into pAV5S vector containing a 98-mer aptamer sequence and as a vector control, aptamer cloned into pAV5S was used to account for any possible RNA-dependent signaling effects (Paige et al., 2011).

Proliferation MTT assays. Cells were grown in 6 cm culture plates until 70% confluence and treated with either PNA3 or control PNA (1μM final) for 24hrs. Next day, plates were trypsinized, counted, 2 X 10^3 cells were seeded into 96-well and MTT assay was performed as previously described (Özeş et al., 2016).

Cell invasion assays. Cells were grown in 6 cm culture plates until 70% confluence and treated with either PNA3 or control PNA (1μM final) for 24hr. Next day, plates were trypsinized, serially diluted and 50,000 cells were seeded inside a matrigel invasion chamber insert (Corning Inc., Corning, NY) in serum free media supplemented with 0.1% BSA. Cells were fixed 48hrs later and analyzed (Özeş et al., 2016).

***In vitro* transcription RNA.** Full length T7-promoter driven HOTAIR and ALU cDNAs were cloned into pcDNA3.1 with a single NHEI restriction site after the transcription stop site. Vector (1μg) was linearized by NHEI digestion followed by PCR cleanup (Qiagen, Hilden, Germany). Eluted DNA was *in vitro* transcribed into RNA according to manufactures protocol (New England Biolabs, Ipswich, MA). The total RNA was purified and DNAseI treated and purified per manufacturers protocol (Qiagen).

Biotinylation, folding, and immunoprecipitation of RNA. Purified RNA (1.67μM) was 3'-biotinylated according to manufacturers protocol (Thermo Scientific). After biotinylation, RNA was purified and folded in folding buffer (100mM KCl, 50mM Tris-HCl, 5mM $MgCl_2$, 0.1mM CaCl2, 0.5mM $ZnCl_2$,) by heating to 100°C for 5 minutes and then snap-cooled on ice for 10 min. 100ng of folded 3' biotinylated ALU or HOTAIR RNA was incubated with individual peptide nucleic acids (PNAs) (5μM final) (Supplementary Table. S2) PNA Bio (Thousand Oaks, CA) in 10μL of 1x folding buffer supplemented with RNAse inhibitor (Thermo Scientific) and bovine serum albumin (5mg BSA) for 30 min at 37°C. Next, streptavidin HRP antibody (Cell Signaling, Danvers, MA) (Supplementary Table S3) was added with 500 μL of binding buffer supplemented with RNAse inhibitor and 10% glycerol (IP Buffer) to each reaction and incubated at 4°C for 1hr. Next, protein A/G plus agarose beads (25μL; Santa Cruz Biotech) were added and placed into 4ºC rotator for 1hr. The beads were washed 3 times with 1x IP Buffer supplemented with RNAsein. Recombinant polycomb repressive complex 2 (PRC2, 0.1nM final, Active Motif, cat # 31387) was added and incubated for 3hr at 4°C on a rotator. After incubation beads were washed 3x with 1X IP buffer supplemented with RNAsein. Samples were then boiled (1x sample buffer, 5 min, 100°C) and run on BioRad precast polyacrylamide gel.

Synthesis of PNAs and pHLIP-antiLinc constructs. The PNAs were purchased (PNA Bio) containing cell-penetrating peptide (CPP) RRRQRRKKR. The pHLIP peptide was purchased from (New England Peptide, Gardner, MA): AAEQNPIYWARYADWLFTTPLLLLDLALLVDADEGT(CNPys)G. pHLIP-antiLinc constructs, were conjugated to the C-terminus of thiolated PNA using a cysteine group derivatized with 3-nitro-2-pyridinesulphenyl (NPys) similar to the recently published report (Cheng et al., 2015). To synthesize pHLIP-antimiR constructs, pHLIP-Cys(NPys) and antiLinc PNA (peptide:PNA 1:1.3) were reacted overnight in the dark in a mixture of DMSO/DMF/0.1 mM KH_2PO_4 pH 4.5 (v/v 3:1:1). The thiolated PNAs used in the study are listed in Supplemental Table S2.

Clonogenic survival and Caspase 3/7 cleavage assays. Cells were grown in 6cm culture plates until 70% confluence and treated with either PNA3 or control PNA (1μM final) for 24 hr. Next day, the plates were trypsinized, counted and 2 X 10^4 cells were seeded into either 6-well or 96-well plates in triplicate. 24 hrs later cells were either not treated or treated with indicated concentrations of CDDP or etoposide for 3hrs, washed with 1X PBS and fresh media was added and incubated at 37°C for 24hrs. Cleaved Caspase 3/7 activity, indicative of apoptosis, was detected according to manufacturers protocol (Promega, Madison, WI). Colonies in 6-well plate were visualized with 5% crystal violet and counted. Percent survival of treated cells was calculated relative to untreated samples.

Aldefluor assay and flow cytometry. ALDH1 enzymatic activity was measured using the Aldefluor assay kit (Stemcell Technologies, Vancouver, Canada) following the manufacturer's instructions and as we have described (Wang et al., 2014). Briefly, 1X10^6 cells were resuspended in aldefluor assay buffer containing the ALDH1 substrate, bodipyaminoacetaldehyde (BAAA) at 1.5mM or 2.4mM, and incubated for 30 minutes at 37 °C. The test ALDH1A1-positive population was gated using control cells incubated under identical conditions with the ALDH inhibitor, diethylamino benzaldehyde (DEAB). Relative increase in Alexa-Fluor 488 signal of the ALDH-positive cells was determined by a FACS Aria II flow cytometer (BD Biosciences, San Jose, CA). For each experiment, 50,000 events were analyzed using Cell Quest software (B-D Biosciences, version 3.3) and relative percentage of ALDH(+) cells was calculated compared to DEAB treated cells.

ChIPNA assay. MCF-7 cells were grown to 80% confluence and then 1μM of biotinylated PNA3 or control PNA was added. 24 hrs later cells were, trypsinized, pelleted, washed with 1X PBS and half of the pellet was fixed with 4% formalin solution. For each sample, nuclei were isolated as previously described (Özeş et al., 2016) then resuspended in nuclei ChIP lysis buffer (50mM Tris-HCl, 250mM NaCl, 0.5% Triton-X, 10% glycerol, 15% formamide), sonicated on HIGH for 30 sec on/30 sec OFF for 1 hr. The lysate was centrifuged (13,000 RPM, 4°C, 10 mins), anti-streptavidin antibody (Supplementary Table S3) was added to the soluble fraction for 2 hrs followed by binding of protein A/G plus agarose beads (Santa Cruz Biotech) for an additional 2 hrs at 4°C. Beads were washed 3 times with wash buffer (100mM KCl, 5mM MgCl2, 50mM Tris-HCl, ad 10% Glycerol) at 4°C and then Proteinase K treated. Nucleic acid was separated with TRIzol and RNA was purified using RNAeasy column (Qiagen). The RNA eluate was treated with DNAseI per manufacturers protocol. RNA isolate (1μL) was used per well for qRT-PCR analysis to confirm lncRNA retrieval. LncRNA FIRRE was used as a negative control, LncRNA ANRIL was used as a positive control.

Mouse xenograft experiments. All animal studies adhered to ethical regulations and protocols approved by the Institutional Animal Care and Use Committee of Indiana University. To assess tumorigenicity of cells, cultured A2780_CR5 cells were washed with PBS trypsinized and counted with trypan blue, re-suspended in 1:1 PBS/matrigel (BD Biosciences) and 2x10^6 cells were injected subcutaneously into the left flank of 3- to 4- week-old female nude athymic mice (BALB/c-nu/nu; Harlan, Indianapolis, IN), as described (Wang et al., 2014; Zhang et al., 2008). Engrafted mice (n=6 per group) were inspected three times per week for tumor appearance by visual observation and palpation. Once tumors were ~200 mm^3, mice were treated with either CDDP (2mg kg^{-1}) or PNA (1mg kg^{-1}) or both CDDP and PNA biweekly for two weeks. Blood samples were collected by puncturing the left lateral saphenous vein with a needle and collected using a capillary tube. Tumor length (l) and width (w) were measured biweekly using digital calipers and tumor volume (v) was calculated as $v=½ \times l \times w^2$. The investigator measuring tumor size was blinded to the treatment groups. Mice were sacrificed when tumor diameter reached 2 cm or at the end of study.

ELISA and cytokine release assays. Conditioned media were prepared by washing culture plates with PBS followed by incubation in serum-free RPMI medium with antibiotics for 48 hr and stored at -80°C. Total cell counts were determined and ELISA was performed using kits and procedures from R&D systems (Minneapolis, MN; Cytokine release assay,) and eBiosciences (San Diego, CA; IL-6 ELISA). The data were normalized to the cell number and reported as fold change. IL-6 release assay was performed 3 times and the cytokine release assay was performed once. ELISA from blood samples was performed by centrifuging total blood (13,000 RPM for 5 mins at 4°C) and serum (100 μL) was added to each well of a 96-well plate.

Luciferase assays. A2780_CR5 cells were seeded in 96-well plates (10^4 cells/well) and transfected with pGL3-E-selectin vector (300 ng construct/transfection). To normalize transfection efficiency, cells were co-transfected with PGL4 Renilla plasmid (100 ng). Twenty-four hours after transfection, cells were treated with PNA3 or Control PNA (1μM) for indicated times. Luciferase activity was analyzed using the Dual Luciferase Reporter Assay System (Promega) and a Thermo Scientific Multilabel Plate Reader.

RNAi. The dsiRNA sequences used targeting human HOTAIR (Sense strand 5'-UUCUAAAUCCGUUCCAUUCCACUGCGA-3', and antisense strand 5'-/5Phos/GCAGUGGAAUGGAACGGAUUUAGAA-3') or negative control RNA targeting GFP (Sense strand 5'-CUACAACAGCCACAACGUC-3', and antisense strand 5'-/5Phos/GACGUUGUGGCUGUUGUAG-3'). DsiRNAs were transfected into cells using Lipofectamine 2000 (Invitrogen; per manufacturer protocol).

Immunoblot analysis. Cells were lysed in RIPA lysis buffer (50mM Tris-HCl, 150mM NaCl, 1mM EDTA, 1% NP-40, 0.5% sodium deoxycholate and 0.1% SDS) and protein (approximately 5-10 mg) was loaded on precast 7.5% TGX gels (BioRad, Hercules, CA), blotting was performed as described previously (Rao et al., 2010) using polyvinylidene difluoride (PVDF) membrane (GE Healthcare, Pittsburg, PA) and antibodies for EZH2, H3, H3K27me3, Streptavidin, and Beta-Tubulin (Supplementary Table S3).

RNA extraction and quantitative RT-PCR (qPCR). RNA was extracted from cell lines and tumors and using RNeasy kit (Qiagen, Venlo, Limburg), cDNA was prepared using MMLV RT system (Promega), and qPCR) was performed with total cDNA and primers for indicated genes and GAPDH or EEF1A as the endogenous control (Supplementary Table S4), using Applied Biosystems 7500 Fast RT-PCR system (Life Technologies, Grand Island, NY) and corresponding software, as we have described (Wang et al., 2014). Primers used for can be found in Supplementary Figure S4.

Immunofluorescence quantification. A2780_CR5 cells were plated on glass slides (50,000 cells/well) and incubated at 37°C, and 24 hrs later incubated with 100nM cy3-pHLIP-PNA3 or cy3-pHLIP-Control PNA for 1 hr at 37°C, washed 3x with 1x PBS. Slides were then prepared according to previously published report (Özeş et al., 2016).

Statistical analysis. All data are presented as mean values ± SD of at least three biological experiments unless otherwise indicated. CDDP IC_{50} values were determined by Prism 6 (GraphPad Software, San Diego, CA), using logarithm normalized sigmoidal dose curve fitting. The estimate variation within each group were similar therefore student's *t*-test was used to statistically analyze the significant difference among different groups by using Prism 4.0 (GraphPad Software). For mouse xenograft study, statistical significance was determined using student two-tailed t-test.

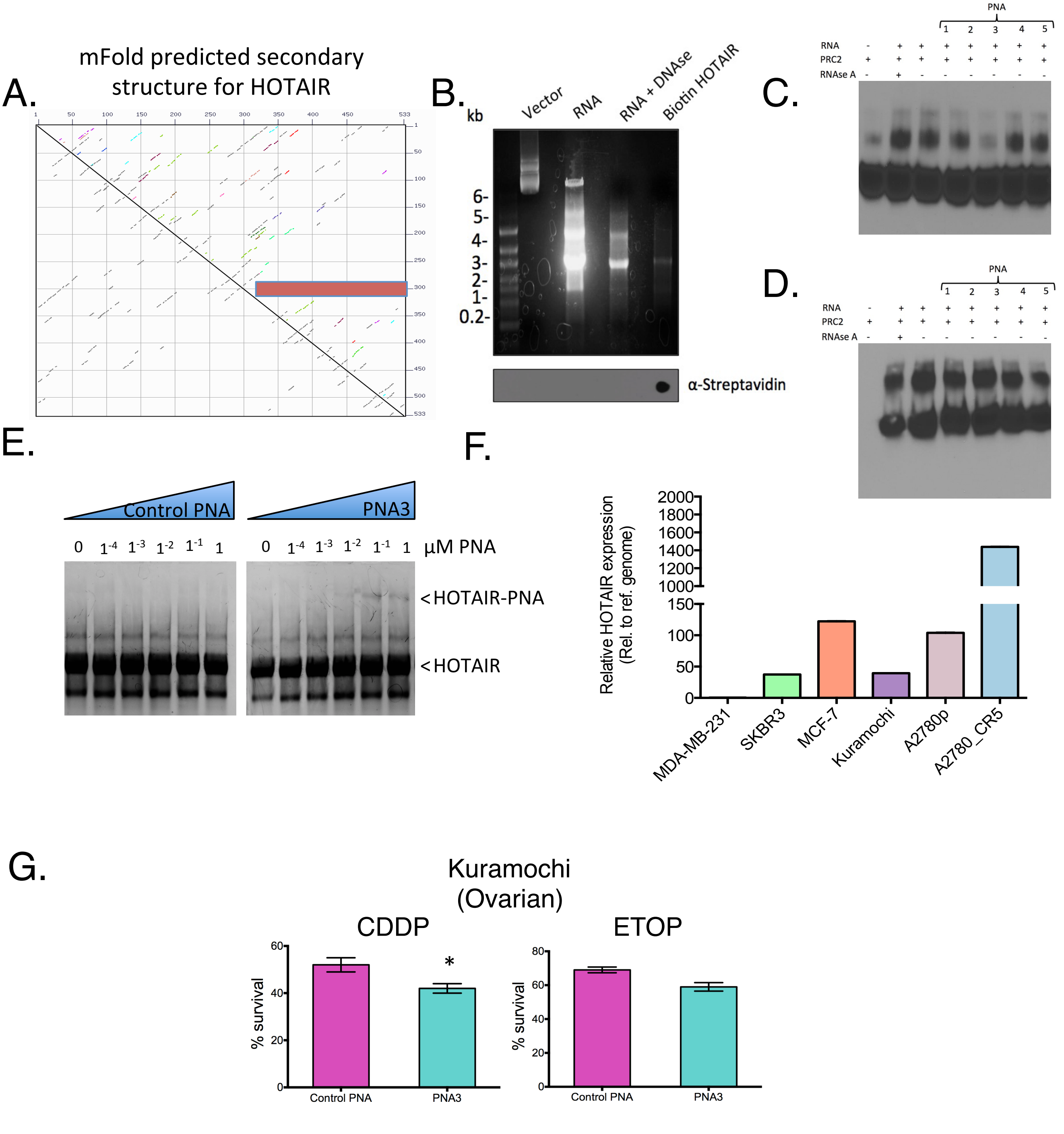

Supplementary Figure S1. **(A)** 2D diagram representing the 19 predicted HOTAIR structures using mFold (red-shaded area corresponds to single-stranded EZH2 validated-binding sites). **(B)** *In vitro* transcription and biotinylation of full-length HOTAIR RNA (uncropped 1% agarose TBE gel). **(C)** Uncropped western showing PNA binding assay with in vitro transcribed HOTAIR and **(D)** ALU RNA **(E)** *In vitro* transcribed HOTAIR (1 μM) was incubated with PNA3 or control PNA (0-1 μM) at 25°C for 1 hr and resolved in a 1%TBE agarose gel. **(F)** Relative HOTAIR expression in breast (SKBR-3, MCF-7, and MDA-MB-231) and ovarian (A2780p, A2780_CR5, and Kuramochi) cancer cell lines. **(G)** Clonogenic survival assays for ovarian cancer cell line Kuramochi.

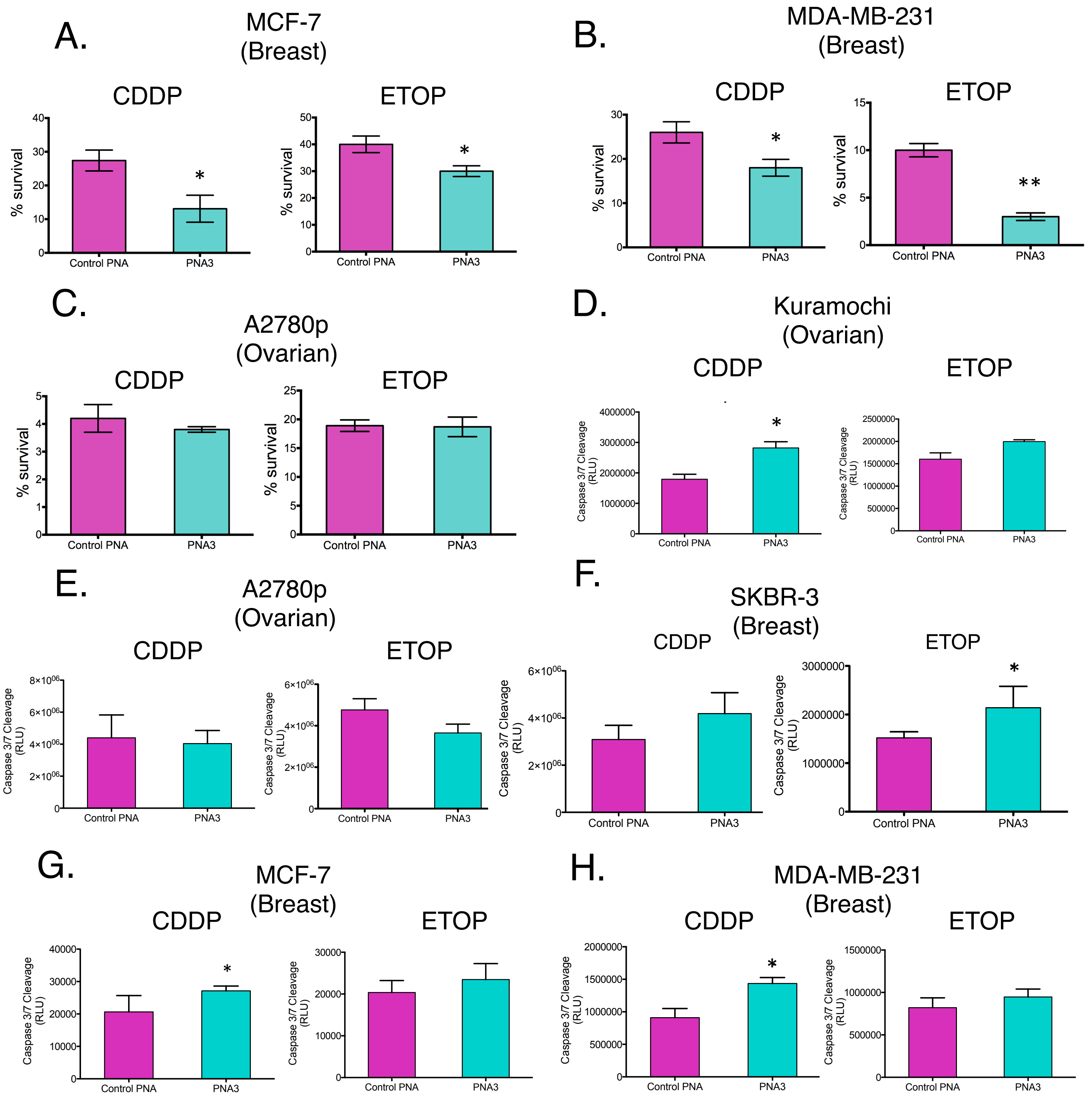

Supplementary Figure S2. Clonogenic survival assays for **(A,B)** breast cancer cell lines (MCF-7 and MDA-MB-231) or **(C)** ovarian cancer cell lines (A2780p) treated with PNA3 (1 μM) or control PNA (1 μM) alone or with chemotherapy (cisplatin (CDDP), 10 μM or etoposide, 5 μM)**.** Caspase 3/7 cleavage assays for **(D,E)** ovarian cancer cell lines (Kuramochi and A2780p) or **(F,G,H)** breast cancer cell lines (SKBR-3. MCF-7 and MDA-MB-231) treated with PNA3 (1 μM) or control PNA (1 μM) and/or chemotherapy (cisplatin (CDDP) (10 μM) or etoposide (5 μM) for 24 hrs). Asterisks indicate P<0.05 (*) or P<0.01 (**).

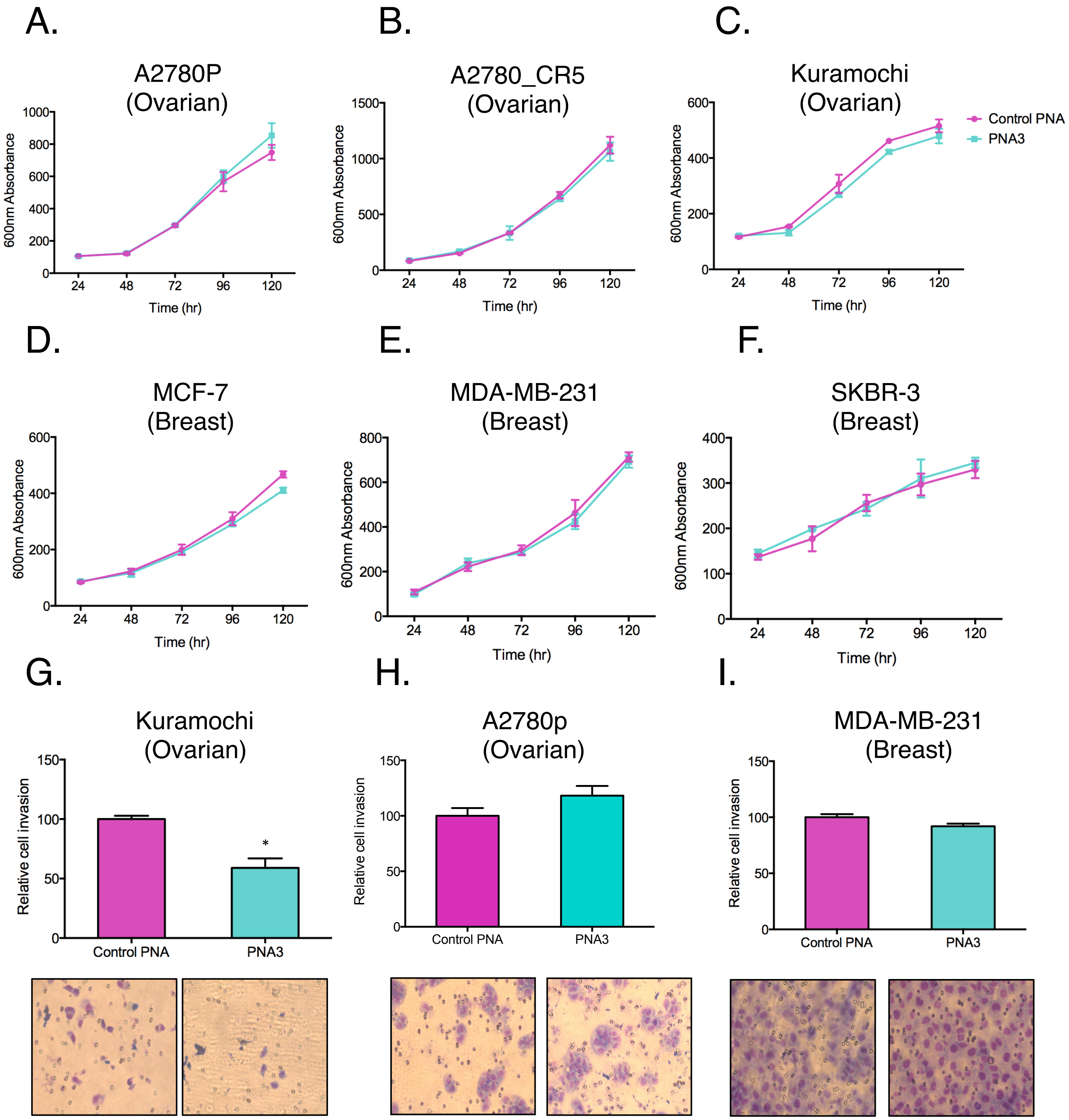

Supplementary Figure S3. Proliferation assays **(A-C)** ovarian cancer (A2780p, A2780_CR5 and Kuramochi) and **(D-F)** breast cancer (MCF-7, MDA-MB-231 and SKBR-3) cell lines treated with PNA3 (1 μM) or control PNA (1 μM) for indicated times and measured by MTT assay. Invasion assays **(G,H)** ovarian cancer (A2780p and Kuramochi) **(I)** breast cancer (MDA-MB-231) cell lines treated with PNA3 (1 μM) or control PNA (1 μM). Cells were counted under 20X magnification and invasion was normalized to control PNA at 24 hrs post seeding. Asterisks indicate P<0.05 (*) or P<0.01 (**).

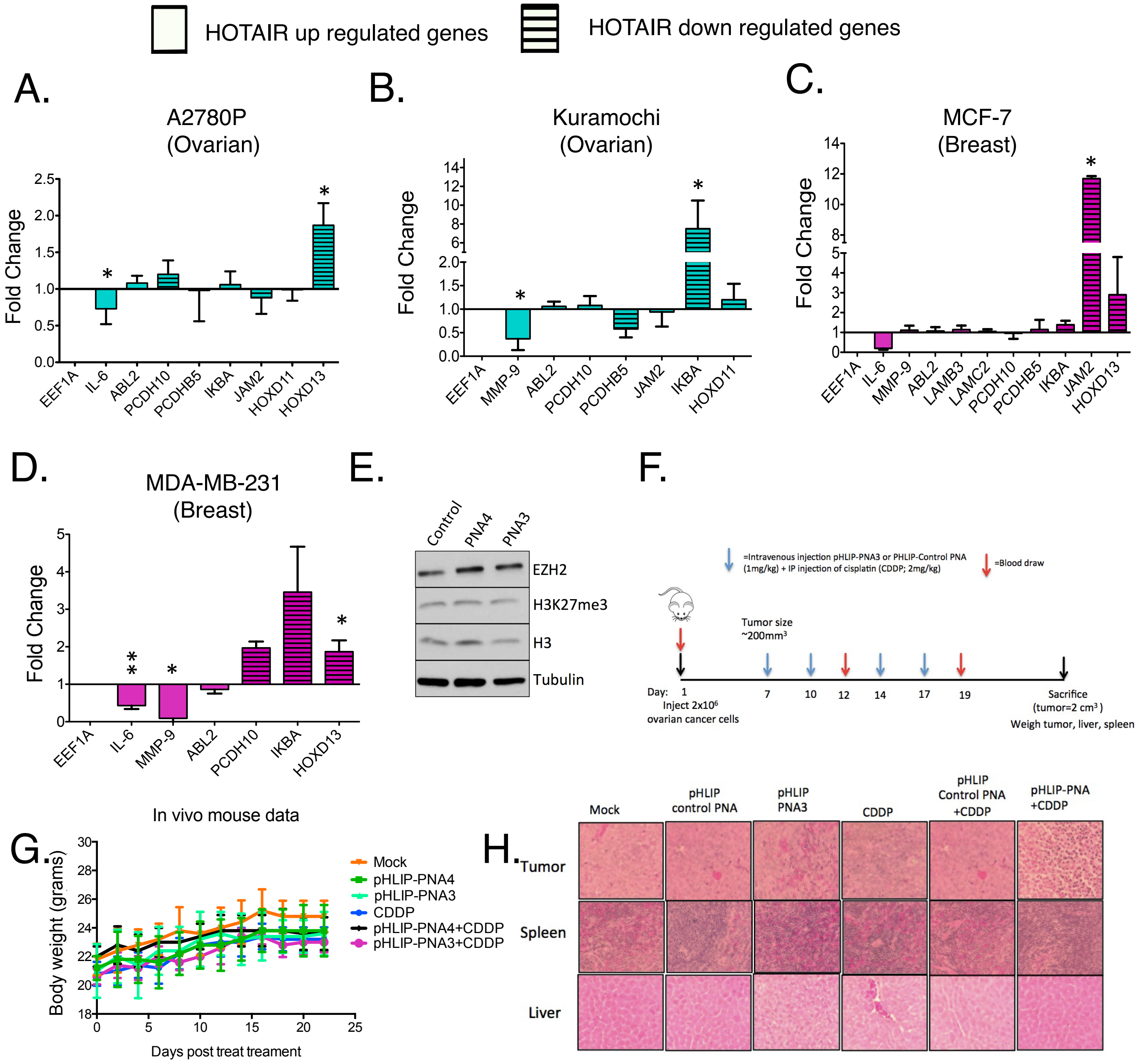

Supplementary Figure S4. HOTAIR target gene expression **(A,B)** ovarian cancer cell lines (A2780p and Kuramochi) and **(C,D)** breast cancer cell lines (MCF-7 and MDA-MB-231) were treated with either PNA3 (1µM) or control PNA (1µM) for 48 hrs. Expression of HOTAIR targets IL-6, MMP-9, ABL-2, LAMB3, LAMC2, PCDH10, PCDHB5, IKBA, JAM2, and HOXD13 was determined by qRT-PCR. **(E)** Total H3k27me3, H3, EZH2 and β-tubulin levels after treatment of in A2780_CR5 cells for 48 hrs with water (control), control PNA (1 µM) or PNA3 (1 µM) and determined by western blot. **(F)** Treatment schedule. Platinum-resistant A2780_CR5 ovarian cancer cells were injected subcutaneously in left flank of each mouse (2X10^6 cells). On day 7, 10, 14 and 16 (blue arrows), mice were treated with vehicle (mock), pHLIP-PNA3 or pHLIP-control PNA4 alone (1 mg kg-1), cisplatin (CDDP; 2mg kg-1), or PNA plus CDDP. Blood was collected on days 1, 12 and 19 (red arrows). **(G)** Body weights over the course of the study were similar am (n=5 per group). **(H)** Representative hematoxylin and eosin (H&E) slides of tumor, spleen, and liver (200X magnification) harvested from endpoint of study; n=3. Asterisks indicate P<0.05 (*) or P<0.01 (**).

Supplementary Table S1. PNA's used in this study

Sense (5'-3')	
**PNAs with cell penetrating peptide**	
PNA1	RRRQRRKKR-TACTGCAGGC
PNA2	RRRQRRKKR -GTAACTCTGGG
PNA3	RRRQRRKKR -TCTGTAACTC
PNA4	RRRQRRKKR -CTCCCCTACTGC
PNA5	RRRQRRKKR -CCCTCTCTCC

Sense (5'-3')	
**Thiolated Cy3 labeled PNAs**	
PNA3	ooo- TCTGTAACTC-ooo-Cys-Cy3
PNA4 (Contr	ooo- CTCCCCTACTGC-ooo-Cys-Cy3

Sense (5'-3')	
**Thiolated PNAs used in in vivo mouse study**	
PNA3	ooo- TCTGTAACTC-ooo-Cys
PNA4 (Contr	ooo- CTCCCCTACTGC-ooo-Cys

Supplementary Table S2. PNA genome alignment

PNA1	Gene targets	% similar
	HOTAIR variants 1,	100
	HOXC11	100
	HOXC12	100

PNA2	Gene Targets	% similar
	HOTAIR variants 1,	100
	HOXC11	100
	HOXC12	100

		% similar
PNA3	Gene targets	
	HOTAIR variants 1,	100
	HOXC11	100
	HOXC12	100
	PRKAR1B	100
	ZHX2	90

		% similar
PNA4	Gene targets	
	HOTAIR variants 1,	100
	HOXC11	100
	HOXC12	100

PNA5	Gene targets	% similar
	HOTAIR variants 1,2, ar	100
	SNAI3	100
	HOXC11	100
	HOXC12	100
	NCOR2	90
	SCARB1	90
	TRPC6	80
	ANGPTL5	80
	OPCML	80
	TTC8	100
	FOXN3	100
	FOXG1	100
	PRKD1	100
	SFXN1	90
	RIMS1	100
	KCNQ5	100
	EPHB2	80
	ATCAY	100
	NMRK2	100
	ETAA1	100
	C1D	100
	NCOA1	80

Supplementary Table S3. Cell line information

Cell type	Cancer Type	Putative histology	TP53 Mutational profile
A2780p	ovarian	malignant adenocarcinoma; endometroid	Wild-type
A2780_CR5	ovarian	malignant adenocarcinoma; endometroid	Wild-type
KURAMOCHI	ovarian	High Grade Serous	Mutant p.D281Y(Hm)
SKBR-3	breast	adenocarcinoma/ER (-) HER2(+)	Mutant p.R175H
MCF-7	breast	ER(+), PR(+), HER2(-)	Wild-type
MDA-MB-231	breast	epithelial adenocarcinoma/ER(-),HER2(-), PR(-)	mutant

Tp53 mutations are shown as heterozygous (het)
or homozygous (Hm) at indicated residues

Supplementary Table S4. Antibodies used

Gene	Company (City, State)	Catalog #	Dilution used
EZH2	Cell Signaling (Danvers, MA)	5246	1/1000
Streptavidin HRP	Cell Signaling (Danvers, MA)	3999	1/5000
H3K27me3	Cell Signaling (Danvers, MA)	9733	1/1000
H3	Active Motif (Carlsbad, CA)	39763	1/1000
β-tubulin	Santa Cruz (Santa Cruz, CA)	sc-55529	1/5000

Secondary Antibody	Company	Catalog #	Dilution used
Goat anti-Rabbit	KPL (Gaithersburg, MD)	474-1506	1/5000
Goat anti-mouse	KPL (Gaithersburg, MD)	474-1806	1/5000

Supplementary Table S5. Primer sequences

genes and controls

<u>qPCR oligos for HOTAIR target genes</u>

	Sense (5'-3')	Antisense (5'-3')
IL6R	TCACTGGGTGCTCAGGAAG	ACCAGCAAGTGCACAGTCC
MMP9	TTGACAGCGACAAGAAGTGG	GCCATTCACGTCGTCCTTAT
NFKBIA	GCAAAATCCTGACCTGGTGT	GCTCGTCCTCTGTGAACTCC
IL6	TACCCCCAGGAGAAGATTCC	TTTTCTGCCAGTGCCTCTTT
GAPDH	CCATCACTGCCACCCAGAAG	CCTTGCCCACAGCCTTGG
EEF1A	GCCCCAGGACACAGAGACTTTATC	CAACACCAGCAGCAACAATCAG
ABL2	TTCCTGGTGCGAGAAAGTG	TACACTTGGGTGCTGGGTAG
LAMB3	ACTATGCTGTGTCCCAGCTC	ACAGCGCTCACAATTTGG
LAMC2	CTGCGAGAAGTGCAAGAATG	ATCCGTGAGCATGTGGAAG
PCDH10	CTAAACACCAGCGAGCAGAG	TGTCTCCATGACCACTGTCC
PCDHB5	GGTGGATGTGAATGACAACG	GAAAGGGGAGATCATTCTGG
JAM2	AGGCCTATGGGTTTTCTGC	CTCCGACCCAGTTTCTTCC
HOXD12	CTCAACTTGAACATGACAGTGC	AATCTGCTGCTTCGTGTAGG
HOXD13	TGGAACAGCCAGGTGTACTG	AGCTGCAGTTTGGTGTAAGG
ALDH1A1	TCCCGTTGGTTATGCTCATTTG	GGAGTTTGCTCTGCTGGTTTGAC
